# The Application and Efficacy of Hyaluronic Acid Fillers for Chin Enhancement and Retrusion Correction: A Systematic Review of Patient-Reported Outcomes

**DOI:** 10.7759/cureus.48807

**Published:** 2023-11-14

**Authors:** Mustafa Qais Muhsin Al-Khafaji, Nawaf Saleh A Althobaiti, Nusaybah Fahad M Alhassani, Zainab Ali H Alnahwi, Wejdan Ahmed Aldawsari, Sadeem Khalid Alquraini, Ather Hassan Abdrabalameer, Faisal Saad S Alharamlah, Abeer Saad Almalki, Naif Abdullah Alotaibi, Abdulaziz Alabdulkarim

**Affiliations:** 1 Department of Plastic and Reconstructive Surgery, Faculty of Medicine, University of Debrecen, Debrecen, HUN; 2 College of Medicine and Surgery, Taif University, Taif, SAU; 3 College of Medicine and Surgery, Ibn Sina National College, Makkah, SAU; 4 College of Medicine and Surgery, King Faisal University, Hofuf, SAU; 5 College of Medicine and Surgery, University of Tabuk, Tabuk, SAU; 6 College of Medicine and Surgery, Imam Mohammad Ibn Saud University, Riyadh, SAU; 7 College of Medicine and Surgery, Imam Abdulrahman Bin Faisal University, Dammam, SAU; 8 College of Dentistry, Imam Abdulrahman Bin Faisal University, Dammam, SAU; 9 Plastic Surgery, Department of Surgery, College of Medicine, Prince Sattam Bin Abdulaziz University, Al Kharj, SAU

**Keywords:** non-surgical management, patient-reported outcome, dermal filler, retrognathia, retruded chin correction, retrusion correction, chin augmentation, chin enhancement, hyaluronic acid filler, hyaluronic acid

## Abstract

A frequent facial abnormality called chin retrusion, also known as retrognathia, can be detrimental to a person's self-esteem and overall face aesthetics. Hyaluronic acid (HA) injections are one non-surgical approach to this problem that may provide individuals seeking chin augmentation with a relatively less invasive and potentially more affordable alternative. The present literature does not provide enough in-depth systematic reviews of the use of HA in chin augmentation. By completing a complete examination of the information that is currently available, this study intends to fill this knowledge gap, supporting physicians and researchers in better comprehending the efficacy and implications of HA in chin augmentation. The safety and success of any esthetic procedure should be made based on the results reported by the patients, including satisfaction and quality of life. Patients need to receive comprehensive surgical instructions from a medical professional to optimize the results of the HA injections for chin enhancement surgery. Regardless of the reported safety of using HA injections, some unwanted side effects have also been recorded. Indeed, healthcare professionals can make more informed decisions and give a patient comprehensive information about the procedure's risks and benefits to the patients. A systematic review was conducted in accordance with the Preferred Reporting Items for Systematic Reviews and Meta-Analyses (PRISMA) guidelines. EMBASE, OVID, and Google Scholar databases were searched up to June 2023. We concentrated on adult patients treated with HA for chin enhancement, and our research was limited to studies conducted in English. A total of 2,738 patients from 24 articles were studied, with 2,259 receiving HA injections for chin augmentation. When applicable, aesthetic outcomes were assessed using scales such as the Global Aesthetic Improvement Scale (GAIS)/FACE-Q and the Galderma Chin Retrusion Scale. Patient satisfaction increased noticeably. Among the studies, some reported complications following HA injection. While three studies found no significant negative effects, one highlighted a major necrotic complication. HA has proven to be an effective and safe alternative to chin augmentation surgery, with the majority of patients showing high satisfaction rates. However, large-scale randomized controlled trials are needed to obtain meaningful results, which will contribute to the further development of non-surgical cosmetic procedures. These studies may facilitate further innovation and refinement of these techniques and potentially expand the application of HA fillers in facial aesthetics.

## Introduction and background

A significant viewpoint that worries many people is that of facial cosmetic operations [1]. The chin aesthetics procedure is one of the crucial facial features because the lower half of the face has an alluring appearance [2]. A variety of factors, including genetics, skeletal deformities, and dental issues can cause chin retractions. Chin retraction is a disorder that affects a person's appearance and functionality. The course of treatment, which may involve a chin implant, an orthodontic procedure, orthognathic surgery, and the use of dermal fillers containing hyaluronic acid (HA), depends on how severe the condition is. Because it gives us the appearance of beauty and youth, a well-contoured jawline with a distinct contour from the mandibular angle to the chin is desired in both men and women. Chin retrusion is an undesirable facial feature that might cause an unpleasant facial look. HA is a natural element in the body that helps preserve the hydration and flexibility of the skin. Recently, using HA fillers has grown in popularity as a proficient way to repair the chin and fix retrusion [3]. HA fillers, when injected into the chin region, can enhance volume while also enhancing the chin's contour and symmetry, giving the face a more harmonious and attractive appearance. The effectiveness of HA fillers for chin enhancement and retrusion correction has not been the subject of a comprehensive review. The goal of this study was to present a thorough analysis of the effectiveness and significance of employing HA for chin augmentation.

## Review

Materials and methods

The Prospective Register of Systematic Reviews (PROSPERO) (ID: CRD42023452008) was used to register this systematic review, and it was carried out in accordance with the Preferred Reporting Items for Systematic Reviews and Meta-Analyses (PRISMA) guidelines [4]. A comprehensive electronic search of the Embase, OVID, and Google Scholar databases was conducted for studies published between the earliest available date and June 2023. The search strategy following the traditional PICOS method of searching electronic databases was developed independently by one of the authors (Mustafa Al-Khafaji), and it was approved by the study team members prior to implementation. Using the medical subject heading (MeSH), a combination of the following keywords was used ("Hyaluronic Acid Fillers" or "Hyaluronic Acid" or "Dermal Fillers" or "Facial Fillers") and ("Chin Enhancement" or "Chin Augmentation") and ("Retrusion Correction" or "Retruded Chin Correction"). 

Eligibility Criteria 

Chin enhancement and retrusion correction studies for adult patients that used only HA fillers and were published in the English language were included. Studies were required to report patient outcomes, case series, or original articles, including randomized controlled trials (RCTs), prospective cohort studies, retrospective cohort studies, and non-randomized non-comparative studies. The primary outcome of interest is patient-reported outcomes, which include satisfaction, aesthetic improvement, and adverse events related to HA filler treatment. Studies that did not report patient-reported outcomes were excluded. Exclusion criteria included studies that investigated other types of fillers or surgical interventions, studies that did not stratify results based on aesthetic procedure, studies including patients below the age of 18, improper methods (reported a meta-analysis/systematic review, economic analysis, animal study, cadaver study, narrative review, or editorial), and studies that reported no outcomes of interest.

*Selection of Articles and Data Extraction* 

Six reviewers independently assessed the titles and abstracts obtained from the search strategy using "Rayyan" software (Rayyan Systems Inc., Cambridge, MA), following the study's eligibility requirements [5]. Following that, the full texts of the selected abstracts were individually evaluated by all authors to determine the final eligibility. When there was disagreement, the reviewers worked together to reach a consensus. This process ensured that only relevant and appropriate studies were included in the final analysis of this research, improving the accuracy and reliability of the study selection. The first author, title, year of publication, journal, study design, country, number of patients, main complaint, treatment sites, age range, mean age, total of HA filler injected, total number of syringes used, brand of filler, touch up injections, follow-up period, complications, patient improvement rate (GAIS) for investigators and subjects, and overall conclusions were extracted from each study. Risk-of-bias assessment tools were used to independently assess the quality of the included studies. Depending on the study's design, various bias tools were used. The revised Cochrane risk-of-bias tool was used for randomized trials, the MINORS tool was used to assess the quality of non-randomized studies or prospective cohort studies, and the Newcastle Ottawa Scale was used for retrospective cohort studies [6,7].

Evaluation of the Statistical Data 

In spite of our attempt at a rudimentary descriptive statistical analysis, the heterogeneity of the included articles and the absence of data in a format conducive to meta-analysis made it unfeasible to conduct a meta-analysis.

Results

Overview of the Literature 

At the beginning of the study, the authors found 382 publications, 344 from Ovid (which included Embase and research papers from the Cochrane Library), and 38 from Google Scholar. After duplicate papers were discarded, the titles and abstracts of 214 different research were examined. After reviewing the entire texts of 68 publications, we found that 24 satisfied the requirements for inclusion or exclusion. Several factors led to the exclusion of a significant number of articles, including the absence of HA information in two, the use of non-HA materials such as botulinum toxin type A and poly-l-lactic acid (PLLA), alongside HA in 15, the absence of specific patient-reported outcomes in 16, the focus on HA injection complications in three, and the failure of the remaining eight articles to specifically address chin enhancement. Between the beginning of time and June 2023, all of the studies that made up this review were published.

The summary of studies (Table 1) includes chin enhancement and chin retrusion correction using HA.

**Table 1:** Overview of all included studies reporting chin augmentation and retrusion correction using HA HA: Hyaluronic Acid; NA: Not Available

Authors	Name of the journal published in	Study design	Country	Age range	Mean age in years, SD	Number of patients treated with HA	(Sex) Male / Female	Ethnicity of patients if mentioned	Chin augmentation or chin retrusion?	Hyaluronic acid product used (BRAND)
Fischer et al. [8]	Journal of Cosmetic Dermatology	Prospective study	Germany	>30	51	84	10 / 74	NA	Chin augmentation	Juvéderm Voluma
Marcus et al. [9]	Plastic and Reconstructive Surgery	Randomized controlled study	The United States	20-73	48.3	107	12 / 95	Predominantly Caucasian	Chin retrusion	Restylane Defyne
Li et al. [10]	Dermatologic Surgery: Official Publication for American Society for Dermatologic Surgery	Prospective study	China	Female ranged from 21 to 45 years, male ranged from 24 to 40 years	Females: 32.26 ± 8.16. Males: 33.00 ± 6.32	38	15 / 23	Chinese	Chin retrusion	Restylane Lyft
Chen et al. [11]	Dermatologic Surgery: Official Publication for American Society for Dermatologic Surgery	Retrospective study	China - Japan	18-52	26.4	326	28 / 298	Chinese-Japanese	Chin augmentation	HA with high elasticity, Brand not mentioned
Sahan et al. [12]	Journal of Cosmetic Dermatology	Retrospective study	Turkey	28.72-46.4	37.56 ± 8.84	50	0 / 50	NA	Chin augmentation	Hyaluronic acid (HA), Brand not mentioned
Bertossi et al. [13]	Aesthetic Plastic Surgery	Retrospective study	Italy	18.7-56	34.5	150	62 / 88	NA	Chin augmentation	Juvéderm Voluma
Braccini et al. [14]	Journal of Cosmetic Dermatology	Randomized control study	France	N/A	54.7	98	6 / 92	NA	Chin augmentation	ART FILLER
Talarico et al. [15]	Dermatologic Surgery: Official Publication for American Society for Dermatologic Surgery	Non-randomized non-comparative study	Brazil	34-76	53.5 ± 8.3	9	0 / 9	Caucasian, Black, Hispanic	Chin augmentation	Emervel (Restylane) Hyaluronic Acid Dermal Filler
Beer et al. [16]	Dermatologic Surgery: Official Publication for American Society for Dermatologic Surgery	Non-randomized non-comparative study	Florida	N/A	48.5	30	4 / 26	Predominantly Caucasian	Chin retrusion	Juvéderm Voluma XC Injectable Gel
Lowe et al. [17]	Dermatologic Surgery	Non-randomized non-comparative study	London, United Kingdom	NA	43.5	8	3 / 5	NA	Chin augmentation	Restylane SubQ
Bertossi et al. [18]	Journal of Cosmetic Dermatology	Retrospective study	Italy	18-57	36.7	83	27 / 56	Caucasian	Chin augmentation	Juvéderm Voluma XC VYC-20L
Mastroluca et al. [19]	Journal of Cosmetic Dermatology	Retrospective study	Italy	23-60	40.9 ± 9.6	40	40 / 0	Caucasian	NA	Juvéderm Volux (VYC-25L)
Bertossi et al. [20]	Aesthetic Surgery Journal	Retrospective study	Italy	20-45	34.4 ± 2.8	30	14 / 16	NA	Chin retrusion	Juvéderm Volux (VYC-25L)
Calvisi et al. [21]	Journal of Cosmetic Dermatology	Retrospective study	Italy	19-71	43.4	91	0 / 91	NA	Chin retrusion	Juvéderm Volux (VYC-25L)
Beer et al. [22]	Dermatologic Surgery: Official Publication for American Society for Dermatologic Surgery	Randomized control study	Florida, New York	22-80	52	192	22 / 170	Predominantly Caucasian	Chin augmentation	Juvéderm Voluma XC VYC-20L
Ogilvie et al. [23]	Dermatologic Surgery: Official Publication for American Society for Dermatologic Surgery	Randomized control study	Germany, France, Netherlands	20-75	46.2	119	9 / 110	Predominantly Caucasian	Chin retrusion	Juvéderm Volux (VYC-25L)
Huang et al. [24]	Journal of Drugs in Dermatology	Non-randomized non-comparative study	Taiwan	27-49	40	84	0 / 84	Asians	Chin retrusion	Restylane Lidocaine - Restylane Lyft Lidocaine
DeLorenzi et al. [25]	Dermatologic Surgery: Official Publication for American Society for Dermatologic Surgery	Prospective study	Canada	(aged above 18)	52	57	2 / 55	Predominantly Caucasian	Chin augmentation	Restylane SubQ
Bae et al. [26]	Dermatologic Surgery: Official Publication for American Society for Dermatologic Surgery	Retrospective study	Seoul, Korea	19-54	29.4	320	0 / 320	Asian women	Chin augmentation	Viscous HA filler, Brand not mentioned
Belmontesi et al. [27]	Aesthetic Surgery Journal	Prospective study	England	26-56	41	11	3 / 8	Predominantly Caucasian	Chin augmentation	Restylane SubQ.
Urdiales-Galves et al. [28]	Journal of Cosmetic Dermatology	Prospective study	Málaga, Castellón, and Bilbao (Spain)	30-60	47.3	30	4 / 26	NA	Chin augmentation	Juvéderm Volux (VYC-25L)
Guo et al. [29]	Plastic and Reconstructive Surgery	Retrospective study	China	Not mentioned	25 ± 4	80	3 / 77	Asian, Caucasian	Chin augmentation	Restylane - Biohyalux -Neuramis- Yvoire- Juvederm
Raspaldo et al. [30]	Journal of Cosmetic and Laser Therapy	Retrospective study	France	26-80	51.27	102	9 / 93	NA	Chin augmentation	Restylane Sub-Q
Ogilvie et al. [31]	Aesthetic Surgery Journal	Prospective study	Germany, France, Netherlands	20-75	46.2	120	10 / 110	Predominantly Caucasian	Chin augmentation	Juvéderm Volux (VYC-25L)

**Figure 1:**
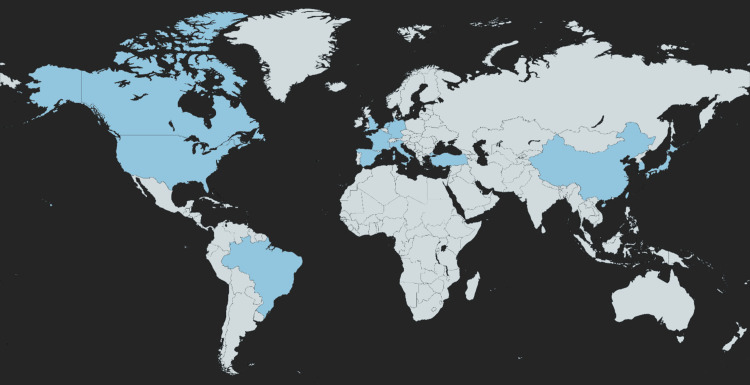
The global geographical representation of research papers regarding chin HA augmentation/retrusion correction The retrieved studies are depicted in blue color.

**Figure 2:**
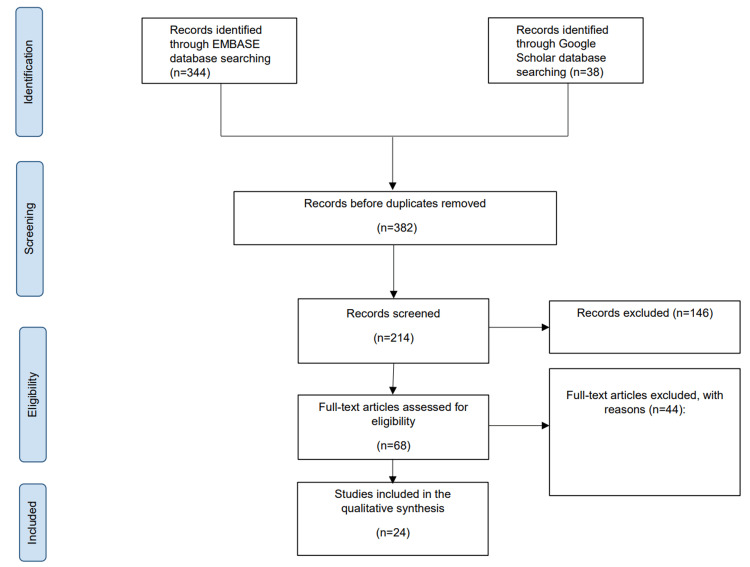
The summary concerning the research studies considered for the purpose of this systematic review according to PRISMA

We looked into four randomized clinical trials, four non-randomized non-comparative studies, six prospective cohort studies, and 10 retrospective studies. There were no case reports or case series that were included. A total of five studies were carried out in Italy, three in the United States, two in the United Kingdom, one in Canada, two in France, two in China, one in Germany, one in Spain, one in Korea, one in Taiwan, one in Turkey, one in both nations of Japan and China, one in Brazil, and two throughout Europe (Germany, France, and the Netherlands), as shown in Figure 1. A synopsis of the PRISMA method is provided for our systematic review in Figure 2.

Patients' Clinical Characteristics

Throughout the entire study, an overall of 2,738 patients have been assessed. This includes 2,259 patients who received HA filler for chin augmentation or treatment of chin retrusion. An explanation of the clinical features of the studies incorporated is available in Table 2. The patients ranged in age from 18 to 80 years old, having a mean age of 42.37. In terms of gender, there were 283 males and 1,976 females (87.5%). The average (initial treatment mean total volume of HA filler injected was 2 mL/patient) ranged between 0.30 and 3.90 mL/patient. In terms of procedural setting, 11 studies were multi-center, four studies were conducted in a private clinic, seven studies took place in the department of plastic surgery in different hospitals, one study took place in a university hospital, and one study was conducted in a research institute. The duration of product efficacy was estimated to range from one to more than 18 months, with a mean of 13 months (12.84). In this review, only six studies reported on the technique of administering (HA). These included the tunneling technique (n=4) and the fanning injection technique (n=2). This review covered any research that utilized HA for chin augmentation and chin retrusion. There are eight research papers that used Restylane, six used Juvéderm Volux, five used Juvéderm Voluma, one used ART FILLER, one study used multiple fillers (such as Restylane, Biohyalux, Neuramis, Yvoire, Juvéderm), and three failed to indicate the kind of HA utilized. The amount of HA provided differed among the research studies examined. Fortunately, 10 studies reported the initially administered volume of HA filler injected, while nine reported the touch-up volumes injected. Meanwhile, 23 of the studies indicated the total amount of HA filler injected mL/patient (initial volume injected + touch-up volume administered). The quantities of injected HA are outlined in Table 2.

**Table 2:** Quantities injected for each study CAF: Case Assessment Form; G: Gauge; SC: Subcutaneous; HA: Hyaluronic Acid; NASHA: Non-Animal Stabilized Hyaluronic Acid; N/A: Not Available

Authors	Hyaluronic acid product used (BRAND)	Initial treatment mean-total of HA filler injected mL/patient	The touch-up volume injected mL/patient	Mean-total of HA filler injected mL/patient	Injection equipment used	Duration of product effectiveness	Injection Protocol (if provided)	Indicate who performed the aesthetic procedure; (Plastic surgeons, Dermatologist or aestheticians)	Where has it taken place, hospital, aesthetic center, plastic surgery clinic	Country
Fischer et al. [8]	Juvéderm Voluma	NA	NA	0.66 mL	Each injector was provided with three syringes of Voluma (2.0 mL) per patient	4 months	Inject Voluma as per normal practice and the CAF was completed posttreatment. Key data collected from the injectors via the CAF comprised type of anesthesia used, site of injection(s) and volume injected, ease of injection, ease of sculpting/massaging, overall cosmetic effect (including smoothness), aesthetic effect versus previous Restylane and preference, probability of recommending Voluma, and details of any adverse events.	Dermatologists, Plastic surgeons, and Aesthetic practitioners [medical doctors and nurses]	Skin and Laser Centre	Germany
Marcus et al. [9]	Restylane Defyne	2.61 ± 0.957 mL	1.35 ± 0.620 mL	3.6 ± 1.419 mL	Utilising a 27G, ½ inch ultra-thin wall needle, Restylane Defyne having 20 mg/ml of hyaluronic acid as well as 3 mg/ml of Lidocaine hydrochloride has been injected into the patient's chin.	3-12 months	A needle is inserted within both of the lines connecting the pre-jowl sulcus and the oral commissures, into the chin and the region inferior to the lower part of the lip. The chin-filling injection technique used by the authors, which involves six to eight injection sites, is an effective technique with a low rate of complications and high patient satisfaction.	NA	Performed at 11 centers	The United States
Li et al. [10]	Restylane Lyft	N/A	Females: 1.89±0.74 mL Males: 2.68±1.28 mL	Females: 1.89±0.74 mL Males: 2.68±1.28 mL	27 gauge sharp needle	12 months	Entry points: The pogonion and the menton. If necessary, an extra entry point was set at the midpoint between the pogonion and menton. A 27G was used for midline chin injections, inserted perpendicular to the skin at each injection site until it reached the periosteum. Although the midline of the chin away from the lower lip area is a vascularized area, it is recommended to inject HA slowly with low pressure after aspiration. The mental ligament was used to achieve local lifting. A blunt-tipped 23G cannula was inserted via this entry point to modify the lower mandibular margin. For male patients, the lateral mental (LM) point (the midpoint of the line between the ML and the gnathion) could be added for chin injection to improve the jaw width	Plastic surgeons	University Hospital	N\A
Chen et al. [11]	HA with high elasticity, Brand not mentioned	NA	0.1-0.3 mL	(median)= 1.85mL	2 different-sized needles were used, for two different purposes	The majority of enhancements lasted up to six months, with partial enhancements remaining visible for up to a year.	First, they started by removing make-up, followed by thoroughly washing off their face. Appliance on topical anaesthetic cream. At the time of the injection, those receiving treatment were in a semirecumbent position. An antiseptic solution for sterilization purposes was used. The chin and surrounding areas were sterilised on three separate occasions using ethanol. A sharp needle was used to inject HA into the subperiosteal plane to increase bone volume and improve chin projection and width. A cannula was used to perform subcutaneous plane fanning and threading injections to smooth the surrounding skin, soothe the pre-jowl sulcus, and decrease the labio-mandibular groove on both sides of the face.	NA	Plastic Surgery Department	China-Japan
Sahan et al. [12]	Hyaluronic acid (HA), Brand not mentioned	NA	0.2-0.4 mL	(median) = 2.25 mL	Using just a single midline entry point, a 25G 50 mm injection cannula was used to inject the hyaluronic acid. A multilayered, retrograde, lateral-to-medial form of tunnelling technique was used to augment the chin both supra-periostally and subcutaneously.	18 months	Sterilisation was done using a topical antiseptic with 2% phenoloxyethanol and 0.1% octenidine hydrochloride. The next step is to find the menton, the chin's most inferior projecting point. A 23G punching needle is inserted through the skin and SC tissues perpendicularly and then removed. This single midline point of entry was used to insert a 25G 50 mm injection cannula, and 20 mg/mL cross-linked HA with 0.3% Lidocaine was added to the suggested augmentation area. Cannula from a single entry point in the middle. A multilayered, retrograde, lateral-to-medial form of tunnelling technique was used to augment the chin both supra-periostally and subcutaneously.	NA	Doctor Al-Sa Aesthetic, Cosmetic and Dermatology Clinic	Turkey
Bertossi et al. [13]	Juvéderm Voluma	1 mL	NA	NA	25–27 Gauge needle	8 months	The patient was positioned upright in the Frankfurt plane before marking to determine the areas to be treated. (Juvederm Voluma) HA was injected above the periosteum and the deep dermal plane using a 25–27 gauge needle. The planned amount of injected filler was 1 mL.	Surgeons	Department of Oral and Maxillofacial Surgery	Italy
Braccini et al. [14]	ART FILLER	NA	NA	(max) = 1.0 mL	27 Gauge 1/2 (13 mm) TSK® needle or a 25 Gauge 55 mm cannula (SoftFil®).	540 days-18 months	The same amount for each product was injected to the subjects The amount of product injected varied according to the deficit to be corrected, but a maximum of 1.2 ml was authorized to be injected for the midface, 1.2 ml for the temples, 1.2 ml for the jawline, and 1.0 ml for the chin via one single supra-periosteal bolus injection with a 27G1/2 (13 mm) TSK® needle or a 25G/55 mm cannula (SoftFil®).	Aesthetic surgeons	Five different centers	France
Talarico et al. [15]	Emervel (Restylane) Hyaluronic Acid Dermal Filler	NA	NA	1.1 ± 0.9 mL	23 G ultrathin wall needle.	18 months	The choice of the injection technique, injection depth (into the supraperiostic zone or subcutis), and injected volume were at the discretion of the investigator.	NA	4 centers	Brazil
Beer et al. [16]	Juvéderm Voluma XC Injectable Gel	1.655 mL	0.6-2 mL at 1 month	2.1 mL	27 Gauge · 1/2”	12 months	The amount of filler used varied depending on the size of the face and the deficit. HA filler was injected into the supra-periosteal plane. Aspiration was done prior to injection.	NA	Research Institute of the Southeast, West Palm Beach, FL	Florida
Lowe et al. [17]	Restylane SubQ	3.9 mL	NA	3.9 mL	Not provided for chin augmentation	12+ months	NA	Dermatologist, Plastic Surgeon	The Cranley Clinic for Dermatology, King Edward VII Hospital	London, United Kingdom
Bertossi et al. [18]	Juvéderm Voluma® XC VYC-20L	NA	NA	1.75 mL for patients over 40 years old, and 1.65 mL for patients under 40 years old.	27 Gauge, 12-mm-long needle	12 months	Supra-periosteal injection using a 27 G needle 12 mm long	Maxillofacial surgeon	Department of Maxillo-Facial Surgery, University of Verona “G.B. Rossi Hospital”	Italy.
Mastroluca et al. [19]	Juvéderm Volux (VYC-25L)	NA	NA	Total 1.7 mL for different sites in the chin (mean is not available for chin only) ranges from 0.2 to 1.0 for each area	A 25 Gauge 38 mm cannula was used for the pre-auricular area, mandibular body, lower pre-jowl, lower anterior chin, labiomental angle, and lateral chin & a 27 Gauge 13 mm needle was preferred for chin apex, anterior chin, pogonion, lateral lower chin, and mandibular angle.	18 months	The standardised MD CodesTM served as the basis for injection points which employed the Volux®. Utilising a linear approach, 0. 5–1 mL of VYC-25 for each side was injected SC into the lower pre-jowl and lower anterior chin in the jaw line. Utilising a fanning technique, labiomental angle was injected SC with 0. 5–1 mL per side. The chin's apex was injected in the middle line, SC, or supraperiosteally with 0. 2–0.4 mL on each side. 0.2–0.4 mL per side were injected into the anterior chin in the supra-periosteal plane, being careful not to get too lateral because of the mental artery. 0.2–0.4 mL was utilised to inject the anterior chin/pogonion SC in the middle line, 0. 2–0.4 mL was utilised to inject the lateral lower chin supraperiostally, and 0. 1–0.5 mL of VYC-25 per side was utilised to inject the lateral chin in the subcutaneous plane using a fanning technique. Due to the high viscosity of VYC-25, the injection regions were lightly massaged using fusidic acid cream for disinfection and to avoid the formation of pseudo-nodules.	NA	3 centers	Italy
Bertossi et al. [20]	Juvéderm Volux (VYC-25L)	NA	NA	Total 1.6mL for different sites in the chin (mean is not available for chin only) ranges from 0.2 to 0.7 for each area	A 27 Gauge 13 mm cannula was used for the chin apex and vertex & a 25 Gauge 5 cm cannula was used for the labiomental sulcus superficial fat compartment. the jawline and marionette lines were injected using a 25 Gauge 5 cm cannula	18 months (results vary from 9-24) depending on the product used	A grid system of horizontal and vertical lines was used to systematize the process of treatment planning and performance. Lines were traced onto individual patients’ faces with skin pencils to allow precise recording of defects and to generate reproducible injection points that could be precisely quantified. specific injection points were designated for female patients and male patients. Other points were used to further redefine the jawline. Using Equivalent MD Codes standardized approach to treatment paper.	NA	Single center, Department of Oral and Maxillofacial Surgery, University of Verona	Italy
Calvisi et al. [21]	Juvéderm Volux (VYC-25L)	NA	NA	3.8 ± 0.8 mL	Injections of the nose were administered using a 27 Gauge needle, and injections of the chin and jawline were made using a 27 Gauge needle or a 25 Gauge cannula	18 months	Injections of the chin and jaw were focused on the pogonion, submental crease, mentum, pre-jowl sulci, jawline, and/or mandibular angle.	NA	2 centers	Italy
Beer et al. [22]	Juvéderm Voluma® XC VYC-20L	2.5 mL	1.2 mL	3.7 mL	A 27 Gauge 1/2 -inch needle was used for supra periosteal and/or subcutaneous injections, 25 Gauge 11⁄2-inch cannulas were permitted for supra periosteal and/or subcutaneous injections	12 months	Needles for subperiosteal and/or subcutaneous injections into the pogonion, menton, and pre-jowl sulci, cannulas were permitted for supra periosteal and/or subcutaneous injections in the menton and prejowl sulci. An appropriate injection volume for the chin and chin area.	NA	Multicenter, skin Associates of South Florida, The Center for Dermatology, Cosmetic & Laser Surgery, k	Florida, New York
Ogilvie et al. [23]	Juvéderm Volux (VYC-25L)	2.28 mL	1.0 mL	3.43 mL	27 gauge 13mm needle	12 months	NA	NA	At 10 sites (6 in Germany, 2 in France, and 2 in the Netherlands)	Germany, France, Netherlands
Huang et al. [24]	Restylane Lidocaine - Restylane Lyft Lidocaine	2.0 mL (max)	1.0 mL	1.6 mL	NA	12 months	NA	NA	2 hospitals in Taiwan	Taiwan
DeLorenzi et al. [25]	Restylane SubQ	2.1 mL	1.0 mL	1.5 mL	(2 mL) was supplied in a 3-mL glass syringe and injected using a sterilized 16-gauge Coleman infiltration cannula (7 or 9 cm in length) with a blunt tip (Byron Medical Inc., Tucson, AZ).	12 months	Sterilization, local anaesthesia, dermal incision. Transdermal insertion of the blunt-tipped cannula for the administration of Restylane sSubQ into SC or Supra periosteal tissue. massage the treatment area to conform to the contour of the surrounding tissue. if necessary.	NA	4 centers in Canada	Canada
Bae et al. [26]	Viscous HA filler, Brand not mentioned	NA	NA	0.5-1 mL	20-mg/mL smooth, highly cohesive, viscous HA filler using a 21 Gauge cannula	10-18 months	A 20-mg/mL smooth and cohesive hyaluronic acid (HA) filler was injected using a 21G cannula to minimize the risk of embolization. The cannula was inserted just above the periosteum, and the filler was slowly injected while withdrawing the cannula through the muscle layer to the subcutaneous plane. The volume of the injection was determined by the patient's condition. After the procedure, a cold compress was applied to reduce swelling and soreness, and dressings were generally not required.	NA	Department of Dermatology. Mary’s Hospital, College of Medicine, Catholic University of Korea. Yangjae Main Center, CNP Skin Laser Clinic	Seoul, Korea
Belmontesi et al. [27]	Restylane SubQ.	1 mL	NA	1 mL	Blunt-tipped 16-gauge cannula	17 months	Dermal incision, transdermal insertion of a blunt-tipped 16G cannula for administration of HA into SC or supra-periosteal tissue. The NASHA gel was injected in small aliquots throughout the area requiring augmentation by manipulating the cannula into a different tract after each injection, using a tunnelling technique.	By Dr. Belmontesi	Multicenter	N\A
Urdiales-Galves et al. [28]	Juvéderm Volux (VYC-25L)	NA	NA	0.2-0.4 mL	A small midline incision is made in the lower chin with a No. 11 blade to facilitate entry of an 18-gauge injection cannula; this is inserted in a lateral direction to sufficient depth to bring the tip into direct contact with the underlying bone	Its biointegration was totaled at day 30 and practically complete at 48 hours of treatment.	Deep tunneling technique along the mandibular bone	NA	3 Aesthetic Spanish Centers	Málaga, Castellón, and Bilbao (Spain)
Guo et al. [29]	Restylane - Biohyalux -Neuramis- Yvoire- Juvederm	≤1 mL OR ≥1 mL	NA	Some patients got ≤1 mL, others got ≥ 1 mL	NA	6 months	NA	NA	Maxillofacial Department of the Chinese Academy of Medical Science and Plastic Surgery Hospital	China
Raspaldo [30]	Restylane Sub-Q	1.9 cc (range 1.2–2cc).	N/A	1.9 cc (range 1.2–2cc).	Voluma was administered via a 19-gauge cannula and by a 21-gauge needle. A dose of 1.9 cc was used to treat chin retrusion.	1-18 months	In order to prevent harming deep facial elements, the piston inside the Voluma syringe was continuously pushed throughout the entire procedure, resulting in the formation of multiple tunnels. The little viscous HA bolus is thick and always comes before the needle tip, acting just like a cushion that helps dissect the tissue prior to the needle. The most effective way to administer Voluma treatment is to cross the various tunnels across various planes in order for the product to spread into and under the fat pads. Any large boluses should be avoided. Towards the end of the procedure, a massage is applied. Voluma gently spreads through every single one of the tunnels, leading to in a harmonious, natural-looking appearance.	NA	1 clinic	France
Ogilvie et al. [31]	Juvéderm Volux (VYC-25L)	NA	NA	3.43 mL	27-guage 13-mm needle was used	Significant improvements following initial Volux treatment in chin projection were achieved at day 30 and generally maintained through 18 months	An injection of a 27G needle was used for the administration of Volux into the chin. A maximum volume of 4.0 mL was allowed for the initial and touch-up treatments combined, and up to 4.0 mL could be injected for repeat treatment. No more than 2.0 mL could be injected into any single treatment area. Treatment areas included the pogonion, mentum, pre-jowl sulci (left and right), and sublabial (mental) crease.	NA	Ten investigational sites in Germany (6 sites), France (2 sites), and the Netherlands (2 sites)	Germany, France, Netherlands

Merely seven studies disclosed the injecting specialist's specialty: two by a dermatologist and a plastic surgeon, two by plastic surgeons solely, two by aesthetic surgeons, and a single one by a maxillofacial surgeon. Throughout all the studies, the cannula sizes utilized for the study participants varied from 16 gauge to 27 gauge, 13 research studies used 27 gauge needles as their preferred option for administering HA. A total of 19 studies injected HA into the intended plane subcutaneously and/or supraperiostally. A total of 0.30-3.90 mL of HA was injected per patient for optimum chin augmentation/retrusion correction, with a mean of 2 mL/patient. In one of studies, Marcus et al. injected 2.61 mL on each occasion until ideal outcomes for the whole chin were attained, and upon the four-week touch-up, he injected 1.35 mL [9]. Moreover, 12 studies used lidocaine as a local anesthetic during HA chin augmentation, and 11 studies included in this review used postoperative antibiotics, anti-inflammatories, and hyaluronidase (if indicated). The aesthetic effects of HA fillers were collected by a particular survey utilizing several scales and instruments, such as the global aesthetic improvement scale (GAIS) / FACE-Q questions or Galderma chin retrusion scale if present for SUBJECTS. Seventeen research investigations found a statistically significant improvement and enhancement in the scale utilised and the overall satisfaction among patients. The overall patient satisfaction using the scales is outlined in Table 3.

**Table 3:** Summary of the overall patient satisfaction using the scales GAIS: Global Aesthetic Improvement Scale; TRAEs: Treatment-Related Adverse Events; HA: Hyaluronic Acid; NA: Not Available

Authors	Patient improvement/response rate (Patient satisfaction)/responder rate	Global Aesthetic Improvement Scale (GAIS)/FACE-Q questions or Galderma Chin Retrusion Scale if present for INVESTIGATORS	Global Aesthetic Improvement Scale (GAIS)/FACE-Q questions or Galderma Chin Retrusion Scale if present for SUBJECTS	Complications if present	Follow-up period
Fischer et al. [8]	Ninety-eight percent of patients rated the overall cosmetic effect of Voluma as very much improved. Treatment was well tolerated.	NA	NA	All adverse events were transient, and the average duration of adverse events was 6.57 days (range: 2–10 days). Four patients suffered bruising, and two patients reported swelling (data missing for one patient).	Not mentioned
Marcus et al. [9]	Aesthetic improvement rates were high throughout the study as reported by subjects. Subject satisfaction was higher in the hyaluronic acid filler Restylane Defyne group than in the control group. In the individual FACE-Q scale items, almost all subjects were satisfied at week 12. Global Aesthetic Improvement Scale (GAIS) also displayed similar results. Treatment-related adverse events were mild to moderate.	The long treatment effect was confirmed by the Global Aesthetic Improvement Scale results, where almost all subjects ≥96 percent according to treating investigators had aesthetic improvement up to the week-48 time point.	The Global Aesthetic Improvement Scale results, which showed that nearly all subjects—85 percent or more—showed aesthetic enhancement up until the 48th week, backed the long-lasting treatment outcome. The Galderma Chin Retrusion Scale demonstrated high effectiveness (81 percent responder rate in the Restylane Defyne group at week 12), and 74 percent of subjects showed statistically significant effects that lasted up to 48 weeks, indicating the long-lasting correction of chin retraction in most subjects. In comparison to the no-treatment control group (35.1) on the 12th week, the FACE-Q Satisfaction with Chin scale, which measures total scores on a 100-point Rasch-transformed scale representing greater satisfaction, demonstrated a significant improvement in subject satisfaction regarding chin appearance following treatment with Restylane Defyne (78.6). At baseline, the mean scores for both groups were similar: 34.6 for the control group and 37.4 for Restylane Defyne. Between week 12 and baseline, there was a statistically significant treatment difference.	The majority of adverse events were mild, with the exception of one moderate event that involved implant-site pain, and their median duration was 4 days. Implant-site bruising (2%), implant-site swelling (2%), and pain (5% of subjects) were the most frequent events. Two instances of implant-site nodules were observed: one that appeared 53 days after treatment and was described as "irregular resorption for the product leading to a 6-mm nodule." It cleared up after 74 days with hyaluronidase treatment, and the other that appeared on the day of treatment and was called a "filler nodule on the chin" resolved following 112 days without any further action. The study did not find any significant adverse events related to treatment. Patients reported events during the four weeks following injection; these were deemed tolerable and resolved for the most part in 14 days. Following initial treatment, the most frequently reported symptoms in the diaries had been tenderness (90%), discomfort and pain (74%), and swelling (74%).	Conducted over 48 weeks
Li et al. [10]	Mental "chin" improvement and patient satisfaction were extremely high with the authors’ injection technique. It can be concluded that chin filling at 6 to 8 injection sites is a good technique with high patient satisfaction and a low complication rate.	GAIS: 1-month post-operation (female): 1.48 ± 0.65 / (male): 1.40 ± 0.55 3 months post-operation (female): 1.91 ± 0.51 / (male): 1.80 ± 0.45	GAIS: 1 mo postoperation (female): 1.83 ± 0.82 / (male): 1.80 ± 0.45 3 mo postoperation (female): 2.17 ± 0.83 / (male): 1.60 ± 0.55	None of the patients reported vascular embolization–related complications.	6 to 12 months of follow-up period
Chen et al. [11]	The shape and contour of the chin were significantly improved in all patients immediately after injection. At the 6-month follow-up, most of the improvements were retained, and partial improvements remained visible 12 months after the injections.	NA	NA	Pain and swelling were reported in 284 individuals (87.1%), ecchymosis in 31 individuals (9.5%), as well as asymmetry in eight individuals (2.5%). Additional risks that include infection, nodule, necrosis, or a witch's chin were not present. Every individual felt satisfied with the outcome of the HA injections.	N/A
Sahan et al. [12]	Patient satisfaction survey revealed that 2 (4%) patients felt neutral, 18 (36%) patients felt satisfied, and 30 (60%) patients felt extremely satisfied with the result of the procedure.	NA	In accordance with GAIS, nine patients (18%) showed improvement, 28 patients (56%) seemed much improved, and 13 patients (26%) seemed very much improved.	Throughout the surgical procedure, none of the patients experienced any discomfort. Slightly mild to moderate erythema and swelling were reported by 48 out of 50 individuals (96%) and went away in a few days. The procedure caused ecchymosis in four (8%) of the patients. Fortunately, skin necrosis had not affected any of the patients.	N/A
Bertossi et al. [13]	Almost all patients treated in our case study showed a stable long-term aesthetic result with patient satisfaction which was better in the HA filler group rather than the surgical and osteotomy groups, for the low post-surgical complication incidence, in general, it was related to the aesthetic result.	NA	NA	Postoperatively, there was no incidence of infections, asymmetry, or granulomas. After 2 months we observed persistent nodules ( 2 mm nodules on 13 patients were treated with massage and 0.2 ml of hyaluronidase)	6 months to 3 years, with an average of 2.3 years.
Braccini et al. [14]	Beneficial effects on volume restoration were maintained 18 months post-injection; injections of Art Filler® Volume were well tolerated by the subjects and showed less acute side effects, which may be explained by a lower induction of inflammation.	GAIS score: Day 21 post-operation: 92% Proportions of subjects with improvement Day 540 post-operation: 36% Proportions of subjects with improvement	GAIS score: Day 21 post-operation: 84% Proportions of subjects with improvement Day 540 post-operation: 36% Proportions of subjects with improvement GACS: The best success was observed on the chin with 73% of subjects for whom a decrease of one grade in the initial (GACS) The Global Aesthetic Clinical Score was still present at Day 540 (18 months).	92 Side effects were associated with patients either injected with Juvéderm® Voluma or ART FILLER® across this study; Erythema (24) with both fillers, Ecchymosis (4) with both fillers, Hematoma (10) with both fillers, Oedema (1) with Juvéderm® Voluma, Dyschromia (1) with Juvéderm® Voluma, Irregularity at palpation (2) with both fillers, Necrosis (1) with Juvéderm® Voluma, Tyndall effect (1) with Juvéderm® Voluma, Spontaneous pain (45) with both fillers, Pain at palpation (3) with both fillers	18 months
Talarico et al. [15]	At 18 months after the last injection, (78.3%) of subjects reported that the treatment had given them more self-esteem and confidence. Most subjects (94.9%) reported being satisfied (25.9%) or very satisfied (69.0%) with the comfort of the injections. All subjects indicated they would recommend the treatment to family/friends and would like to receive the treatment again. At 18 months after the last injection, (98.3%) of subjects were satisfied or very satisfied with the durability of the results.	Investigators judged full-face GAIS to be improved for all subjects at 3 weeks after the last injection, which persisted after 18 months (95.0%)	3 weeks post-operation: 63% of patients Very much Improved. 33.3% of patients Much improved. 3.7% of patients Improved. 18 months post-operation: 7.4% of patients Very much Improved. 55.6% of patients Much improved. 25.9% of patients Improved.	Only 1 subject (1.6%) experienced 6 bruises limited to the injection site at injections and resolved without treatment before the next visit 3 weeks later. There were no severe AE or AEs leading to study discontinuation. Throughout the next 3 weeks post-injections; 50% of subjects experienced swelling, and 25% experienced bruising. no erythema or pruritus was experienced. The pain worst score was 1.0 ± 1.2	18 months after the last injection
Beer et al. [16]	Mean facial angle significantly improved at all time points compared with baseline, improving by 1.83 at 12 months. Subject satisfaction with lower face and jawline increased significantly for all FACE-Q questions and time points compared with baseline. Subject satisfaction with overall facial appearance and percentage of subjects “not bothered” by the area under the chin increased significantly for most FACE-Q questions and time points compared with baseline.	At 2 months, more than 90% stated much improved. Around 80% stated much improved at 12 months post-injection. At 2 months, only 10 % stated improved. Around 20% stated improved at 12 months post-injection.	When compared to the baseline, patient satisfaction regarding their lower face and jawline raised substantially throughout all FACE-Q questions as well as time points.	Two patients (6.7%) experienced device-related TRAEs, two patients (6.7%) experienced six severe complications unrelated to the device or procedure, and 16 patients (53.3%) experienced 27 treatment-related adverse events (TRAEs). Tenderness to touch (21 patients; 70%), firmness (18 patients; 60%), and lumps/bumps (15 patients; 50%) were the most commonly reported injection-related events. Of the three subjects who reported serious injection-related events, two of them had bruises, and one of them had redness. The majority of injection-related side effects subsided after seven days, but two individuals had lumps or bumps that persisted for more than thirty days. At the final 12-month visit, only one patient reported being bothered by the scars, and only four patients reported being bothered by parts of their face that were not smooth.	12 months
Lowe et al. [17]	All injected patients were satisfied	NA	NA	Hematoma, swelling	4-64 Weeks
Bertossi et al. [18]	83 (100%) patients performed chin augmentation and were satisfied by the results. The average advancement of the chin was 3 mm. There was an improvement in esthetic and psychologic metrics perceived by the patient	NA	Patients complete the FACE Q questionnaire; comparing the scores found before and 90 days after the treatment, we can observe that post‐treatment scores were significantly lower than pre‐treatment scores (P < 0.05). This indicated improvements in esthetic and psychologic metrics perceived by the patient	The overall rate of complications was 1.4%. Asymmetry (0.3%), Bruising (6%), and Mild pain (12%) disappeared in 1.5‐2.5 hours. No cases of skin necrosis or skin loss were observed. None of our patients reported distress.	3 and 8 months
Mastroluca et al. [19]	Patient satisfaction was high	NA	Patient satisfaction was high, as assessed using the FACE-Q ‘Satisfaction with outcome’ questionnaire (mean Rasch-transformed score: 88.1 ± 10.3).	In 40 patients only 20 had complications; soft-tissue edema (12), hematoma (6), and telangiectasia (2). All were early, transient, and minor; there were no major or delayed events.	1 and 6 months post-treatment.
Bertossi et al. [20]	Patient satisfaction with treatment was high	NA	29 patients (96.7%) rated their appearance at 20 days post-treatment as ‘much improved’ or ‘very much improved	Early transient soft-tissue edema and bruising	3 days, 1 week,1 month and 6 months
Calvisi et al. [21]	N/A	NA	NA	Bruising, pain	up to 12 months
Beer et al. [22]	There was a significant improvement in the FACE-Q Satisfaction with Chin at baseline compared to at 6 months	GAIS score: 87.3%	GAIS score: 91.2% at 12 months The FACE-Q Satisfaction with Chin overall mean score for the treatment group was 34.9 at baseline and improved by a mean of 35.6 to a score of 71.3 at Month 6	One participant experienced facial cellulitis and injection site inflammation	12 months
Ogilvie et al. [23]	VYC-25L treatment also led to improved subject satisfaction with the chin and psychological well-being. Importantly, these objective and subjective	GAIS score: 100% at 3 months / 82.1 % at 12 months	GAIS score: 91.8 % at 3 months / 77.2 % at 12 months	NA	12 months
Huang et al. [24]	N/A	GAIS score: 82% at 24 months	GAIS score: 93% at 24 months	Bruising	24 months
DeLorenzi et al. [25]	Patient- and investigator-assessed treatment response rates were declining over 12 months	At 1, 3, and 6 months post-treatment, GAIS score: 100%, 100%, and 96%	at 1, 3, and 6 months post-treatment, GAIS score: 100%, 96%, and 91%	The most commonly reported events were local injection-site reactions such as swelling, tenderness or redness, bruising,	Follow up was 4weeks -6-9-12 months
Bae et al. [26]	Almost all of the patients were very satisfied with this volumizing procedure	GAIS score: 97.2%	GAIS score: 94%	NA	Before 4 weeks after the injection.
Belmontesi et al. [27]	Patient-assessed evaluation of aesthetic outcome at 3 months after treatment with Restylane SubQ indicated that 5 patients were very much improved in appearance, 3 patients were moderately improved	NA	NA	Local swelling, tenderness and redness, bruising, and moderate injection-site pain	3 months
Urdiales-Galves et al. [28]	VYC-25L has been successfully and safely used for volumizing and contouring the chin and jaw area. Additionally, patient satisfaction was really very high, with 90% of patients being “Very satisfied” or “Satisfied” with the treatment results with only 3 patients “dissatisfied” with the treatment results	NA	NA	Late complications associated with the use of dermal fillers, including injection site reactions (erythema, edema, pain/tenderness, etc); infection (erythema, edema, nodule/access, etc); hypersensitivity	Follow-up 48 h and 30 days after treatment
Guo et al. [29]	32 patients (45.71 percent)felt very satisfied, 37 patients (52.86 percent) selected satisfied, and one patient (1.43 percent) reported being dissatisfied because of infection after the injection that was addressed by hyaluronidase injection	NA	NA	Vascular occlusion	NA
Raspaldo et al. [30]	Voluma injectable HA sub-dermal facial filler treatment results in clear aesthetic improvements and increased self-esteem and greater confidence in the majority of patients	GAIS score: 6-18 months 65 patients (64%) were still considered as ‘very much improved’, 17 patients (17%) were rated as ‘much improved’ and 18 patients (18%) were considered ‘improved’. Only one patient (1%) was judged as ‘no change’	GAIS score: 6-18 months ‘very good’ (71 patients [70%]) or ‘good’ (29 patients [28%]	One case of swelling, three cases of hematoma, four cases of overcorrection, and one case of hypersensitivity.	6-18 months
Ogilvie et al. [31]	Overall mean Satisfaction with Chin scores improved from 41.4 (38.6, 44.3) at baseline to 55.1 (51.4, 58.8) at month 18 after initial treatment and 74.2 (69.2, 79.2) after repeat treatment in the Volux group; results were similar in the control group after treatment	GAIS score: 1 month (100-96.2%) 18 months (52.5 -60%)	GAIS score: 1 month (94-92.2%), 18 months (62-64%)	Firmness, redness, pain after injection, lumps/bumps, discoloration, itching.	18 months

Seventeen of the studies encountered complications resulting from HA chin injections. Three studies found no significant treatment-related adverse events, while a single study reported one major complication of necrosis using Juvéderm. In regard to minor complications, 17 studies reported cases of bruising and hematoma, 14 studies experienced swelling and edema, 11 had pain and tenderness at the injection site, eight studies reported erythema and and redness, and five reported nodules with lumps/bumps that were resolved with hyaluronidase. Three studies reported scars and asymmetry, two studies reported pruritus and itching, two studies reported hypersensitivity, one experienced local mobility of the implant, one study experienced overcorrection, and one reported vascular occlusion. The reported duration of follow-up in every study varied from two days to a period of 24 months.

Quality Assessment and Risk of Bias

Two members of the review team (WA and ZA) independently assessed the risk of bias in each study, using the Cochrane Collaboration’s tool for risk of bias assessment in RCTs, three of the included RCTs were considered low risk of bias, and only one was considered high risk (Table 4). Four of the non-randomized non-comparative studies were assessed by MINORS tools, and the total score ranged from nine to 15, with a mean score of 11.9 (Table 5). The Newcastle Ottawa Scale was used to assess bias in 16 of the retrospective, along with prospective cohort research studies, and scored seven out of nine, indicating a high level of quality (Table 6).

**Table 4:** Review authors' judgments about each risk of bias item for each included study RoB: Risk of Bias

	Bias arising from the randomization process	Bias due to deviations from intended interventions	Bias due to missing outcome data	Bias in the measurement of the outcome	Bias in the selection of the reported result	Overall RoB
Marcus et al. 2021 [9]	Low	Low	Low	Low	Low	Low
Braccini et al. 2023 [14]	High	Low	Unclear	Low	Low	High
Beer et al. 2021 [22]	Low	Low	Low	Low	Low	Low
Ogilvie et al. 2019 [23]	Low	Low	Low	Low	Low	Low

**Table 5:** MINORS assessment tool for non-randomized non-comparative studies (n=4)

Item	Talarico et al. 2015 [15]	Beer et al. 2020 [16]	Lowe et al. 2006 [17]	Huang et al. 2020 [24]
A clearly stated aim	2	2	1	2
Inclusion of patients	2	2	1	2
Prospective collection of data	2	2	1	2
Endpoints appropriate to the aim of the study	2	2	2	2
Unbiased assessment of the study endpoint	0	0	0	2
Follow-up period appropriate to the aim of the study	2	2	2	2
Loss to follow-up less than 5%	2	0	2	1
Prospective calculation of the study size	0	0	0	2
Total Score	12	10	9	15

**Table 6:** Newcastle-Ottawa scale for the included cohort prospective and retrospective studies (n=16)

	Selection				Comparability	Outcome			Quality Score	Risk of Bias (0-3: High, 4-6: Moderate, 7-9: Low)
Article	Q1	Q2	Q3	Q4	Q5	Q6	Q7	Q8		
Chen et al. 2022 [11]	*	No description of the derivation of the nonexposed cohort	*	*	No comparison group	*	*	No statement	5	Moderate
Bertossi et al. 2015 [13]	*	*	*	*	*	*	*	No statement	7	Low
Bertossi et al. 2019 [18]	Selected group of users	*	No description	*	*	*	*	*	6	Moderate
Mastroluca et al. 2021 [19]	*	No description of the derivation of the nonexposed cohort	*	*	No comparison group	*	*	No statement	5	Moderate
Bertossi et al. 2021 [20]	*	No description of the derivation of the nonexposed cohort	*	*	No comparison group	*	*	No statement	5	Moderate
Calvisi et al. 2022 [21]	*	No description of the derivation of the nonexposed cohort	No description	*	No comparison group	*	*	No statement	4	Moderate
Bae et al. 2013 [26]	No description of the derivation of the cohort	No description of the derivation of the cohort	No description	*	No comparison group	*	No	No statement	2	High
Guo et al. 2020 [29]	*	*	*	*	**	*	No	No statement	7	Low
Ogilvie et al. 2020 [31]	No description of the derivation of the cohort	No description of the derivation of the cohort	No description	*	*	*	*	*	5	Moderate
Sahan et al. 2020 [12]	No description of the derivation of the cohort	No description of the derivation of the nonexposed cohort	*	*	No comparison group	*	*	*	5	Moderate
Urdiales-Galves et al. 2021 [28]	No description of the derivation of the cohort	No description of the derivation of the nonexposed cohort	*	*	No comparison group	*	No	No statement	3	High
Raspaldo et al. 2008 [30]	No description of the derivation of the cohort	No description of the derivation of the nonexposed cohort	*	*	No comparison group	*	*	*	5	Moderate
Fischer et al. 2010 [8]	No description of the derivation of the cohort	No description of the derivation of the nonexposed cohort	*	*	No comparison group	*	*	No statement	5	Moderate
Li et al. 2023 [10]	No description of the derivation of the cohort	No description of the derivation of the nonexposed cohort	*	*	Study controls for sex (no comparison group)	*	*	*	4	Moderate
Delorenzi et al. 2009 [25]	No description of the derivation of the cohort	No description of the derivation of the nonexposed cohort	*	*	No comparison group	*	*	*	5	Moderate
Belmontesi et al. 2006 [27]	No description of the derivation of the cohort	No description of the derivation of the nonexposed cohort	*	*	No comparison group	*	*	*	5	Moderate
Selection: Q1. Representativeness of the exposure cohort? Q2. Selection of the non-exposure cohort? Q3. Ascertainment of exposure? Q4. Demonstration that outcome of interest was not present at the start of the study? Comparability: Q5. Comparability of cohort on the basis of the design or analysis? Outcome: Q6. Assessment of outcome? Q7. Was follow-up long enough for outcomes to occur? Q8. Adequacy of follow-up of cohorts?										

Quantitative Data Analysis

Since there were differences in techniques, treatments, inadequate extracted data, and outcomes assessed, quantitative meta-analyses were not possible.

Discussion

The primary objective of our systematic review was to gather application and safety data for HA fillers in patients seeking to correct volume deficit and retrusion in the chin. The findings of our review suggest that HA injectable fillers have an acceptable safety profile and are effective for restoring and creating facial volume in contouring the chin and projecting and sculpting it. In addition, the repeated treatment of several facial indications showed a satisfactory safety profile. The chin is a crucial facial feature for overall attractiveness, impacting facial symmetry and balance [32,33]. A rather frequent disorder, chin retraction, can be brought on by heredity, bone anomalies, habitual posture, and dental issues. A common facial abnormality called chin retrusion can make the face appear imbalanced and less attractive [31]. In recent years, using HA fillers to augment the chin and correct retrusion has been popular and effective [34]. A naturally occurring substance in the body called HA helps keep the skin supple and hydrated [35]. When injected there, HA fillers can add volume and improve the symmetry and contour of the chin, giving the face a more beautiful and harmonious appearance. Through reviewing the included studies, most of the patients in all studies' satisfaction rates were exceptionally high, as well as experienced minor and tolerated complications compared to the perfect results of HA filler injection. Reported complications included the following: bruising and hematoma, swelling and edema, erythema and redness, pain, and tenderness in the injected area. All reported complications did not affect the process of injection. Necrosis was reported in one study where an individual used Juvéderm [14]. Contrary to alternative surgical treatments for chin augmentation, the low rate of problems demonstrates that HA injectable fillers are an excellent choice for assuring patient happiness and comfort. These results emphasize the need to use specialized methods to increase the security and effectiveness of HA filler injections. According to GAIS, patients were significantly improved in 13 of the studies under consideration. Several restrictions should be taken into account when evaluating the results of this systematic review. There is a chance of linguistic bias because the inclusion criteria only apply to papers written in English. The exclusion of other papers published in any other language may have compromised the thoroughness of this evaluation.

## Conclusions

In conclusion, research findings suggest that HA injections could serve as a suitable and effective treatment to enhance the overall appearance of an individual's chin. Through this endeavor of performing a systematic review, we intend to demonstrate through our analysis greater patient satisfaction, long-term aesthetic effects, and product efficacy for up to 18 months post-treatment. Apart from one serious event that was not directly associated with HA injection administration, the problems reported seemed to be mostly minor. Injectable chin HA offers a special therapeutic and cosmetic potential for treating patients unable to have surgery, thus providing a viable and beneficial option that has the potential to be used to address a range of chin aesthetic and functional challenges as part of the plastic surgeon's reconstructive and aesthetic choice. HA is clearly influential, and we consider it a safe and great alternative to surgical treatments, with the degree of retrusion determining the treatment option. Larger research, however, is required to get more definitive findings.
